# Effect of bile salts on intestinal epithelial function in gilthead seabream (*Sparus aurata*)

**DOI:** 10.1007/s10695-024-01369-8

**Published:** 2024-06-25

**Authors:** J. Fuentes, S. F. Gregório, F. Fonseca, R. Robles-Arozarena, J. A. Martos-Sitcha, F. J. Moyano

**Affiliations:** 1 Consejo Superior de Investigaciones Científicas (ICMAN-CSIC), Instituto de Ciencias Marinas de Andalucía, 11519 Puerto Real, Cádiz, Spain; 2 Centro de Ciências do Mar, Universidade do Algarve, 8005-139 Faro, Portugal; 3 ARNET (Aquatic Network, Associated Laboratory), Centre for Marine and Environmental Research (CIMA), University of Algarve, Faro, Portugal; 4 Testing Blue S.L., Calle Holanda 26, 11500 Puerto Real, Cádiz, Spain; 5 Departamento de Biología Facultad de Ciencias del Mar y Ambientales, Instituto Universitario de Investigación Marina (INMAR), Campus de Excelencia Internacional del Mar (CEI-MAR), University of Cádiz, 11519 Puerto Real, Cádiz, Spain; 6 Departamento de Biologia y Geologia Facultad de Ciencias, Campus de Excelencia Internacional del Mar (CEI-MAR), Universidad de Almeria, La Cañada de San Urbano, 04120 Almería, Spain

**Keywords:** Sea bream, Intestine, Ussing chamber, Bile salts, Intestine

## Abstract

In the context of modern aquaculture, the effort to reduce the reliance on fishmeal/marine ingredients in fish diets has led to the exploration of plant-based protein sources as potential substitutes, a dietary shift that disrupts the bile acid profile in fish. Therefore, bile salts are being sought as additives. However, artificially increased intestinal levels of bile acids may significantly impact mucosal function. Therefore, here, we explored the regulatory role in the intestine of gilthead sea bream (*Sparus aurata*) of (i) chenodeoxycholic acid (CDC), (ii) a mixture formed by two bile acids, 3% cholic acid and 97% deoxycholic acid (MIX), and (iii) a conjugated bile salt sodium taurocholate (TC) in Ussing chambers with the epithelial voltage clamp technique. We tested the bile salts in a 50–500 μg/ml concentration range, and all of them promoted ion absorption. Yet, clear concentration-dependent and more pronounced effects on the ion transport were observed in the posterior intestine. On the other hand, bile salts had no or minor effects on tissue resistance. However, there are indications that the MIX could have adverse effects at high concentrations (500 μg/ml), promoting a threefold increase in tissue permeability measured using FITC-dextran (4 kD) regardless of the intestinal region, thus suggesting an alteration in intestinal permeability at high bile salt concentrations. The findings from our study emphasize the importance of considering intestinal function when contemplating the possible use of a particular bile salt as a dietary supplement. It appears that bile salts, whether acting individually or in combination, play a pivotal role in orchestrating nutrient absorption by influencing the function of epithelial ion transport. However further research is needed to fully grasp the region-dependent nuances of bile salt effects on ion transport and the ultimate consequences for nutrient absorption in the context of fish aquaculture.

**Supplementary Information:**

The online version contains supplementary material available at 10.1007/s10695-024-01369-8.

## Introduction

Bile acids are classically known for their roles in facilitating the digestion of fats, although many other functions have been acknowledged in the last few years. They act as regulators of epithelial homeostasis, transport, and barrier function at the intestinal level in vertebrates (Hegyi et al. 2018) and also support antimicrobial activities that can influence the gut microbiome (Hagey et al. 2010). Moreover, bile acids can act as pro-inflammatory agents that activate nuclear receptors and cell signaling pathways to regulate lipid, glucose, and energy metabolism (Chiang 2009). Additionally, in the case of fish, there is some evidence that bile acids act as pheromones (Giaquinto and Hara 2008), affect taste stimuli (Rolen and Caprio 2008), and likely function in inter- and intraspecific communication (Zhang et al. 2001).

In the current aquaculture, efforts to reduce the reliance on fishmeal in fish diets have led to the exploration of plant-based protein sources as potential substitutes. This dietary shift can have repercussions on digestion, an often-overlooked facet. Plant protein-based diets typically have lower taurine levels, which is highly present in fishmeal (Aragão et al. 2014). Taurine plays a critical role in the conjugation of bile acids in fish (Hofmann et al. 2010). Therefore, feeding fish with diets deficient in taurine results in diminished bile salt concentrations due to a reduction in its availability, as shown in Korean rockfish *Sebastes schlegelii* (Kim et al. 2015). Also, low dietary levels of taurine may impair fat digestion indirectly considering that taurocholate is the primary salt present in fish bile (Goto et al. 1996; Kortner et al. 2014) and that taurine-conjugated bile acids are more efficient fat emulsifiers than glycine-conjugated bile acids (Goto et al. 1993; Chesney et al. 1998). From a strictly practical point of view, there is evidence that using plant protein meals in diets for some aquacultured fish is often associated with decreased lipid digestibility, reduced bile acid levels, and hypocholesterolemia (Krogdahl et al. 2010; Tocher et al. 2008; Francis et al. 2001). Using such ingredients disrupts the bile acid status, resulting in increased excretion/decreased intestinal reabsorption and modified bile acid synthesis (Staessen et al. 2020; Kortner et al. 2014). As detailed in a recent review by Romano et al. (2020), there is an association between impaired bile acid transport and intestinal inflammation induced by several anti-nutritional factors like saponins, oligosaccharides, high molecular mass proteins, fibers, and specific amino acid residues that may bind bile salts preventing their reabsorption. Furthermore, low contents of cholesterol and the absence of taurine in plant protein sources are likely to reduce bile acid synthesis.

Bile salts are shown to be capable of reducing or mitigating the effects of plant-based diets. In Sparids, bile acid supplementation showed growth-promoting effects in the sea bream (*Sparus aurata*) at levels of 0.06–0.12% (Ruiz et al. 2023a), but not in the black sea bream (*Acanthopagrus schlegelii*) at levels of 200 mg/kg of feed (Jin et al. 2019), with benefits to integrity and immune responses in both species. In rainbow trout (*Oncorhynchus mykiss*), dietary supplementation with bovine bile acids (1.5%) or synthetic taurocholate (1%) prevents decreased growth and distal intestine and liver abnormalities caused by soybean meal or soy antinutrients (Iwashita et al. 2008, 2007; Yamamoto et al. 2007). In contrast, neither purified taurocholate nor bovine bile salt (at 1.8% supplementation) could revert the poor growth performance, lipid digestibility, and intestinal inflammation caused by dietary plant protein in Atlantic salmon (Kortner et al. 2016). Dietary supplementation with taurocholate attenuated but did not completely counteract soybean meal-induced enteritis in *Scophthalmus maximus* (Gu et al., 2016). These observations point to a possible negative interaction of the supplementary bile salts with some plant compounds like lectins or flavonoids that neutralize their potential beneficial effect.

On the other hand, artificially increased intestinal levels of bile acids may significantly impact mucosal function. Studies in humans demonstrate that low concentrations of bile acids stimulate enterocyte proliferation, while higher concentrations cause cell death. This effect is closely related to the typology of the bile acid, i.e., conjugated, hydrophilic, deconjugated or dehydroxylated (Yui et al. 2009; Katona et al. 2009). Also, it is well established that exposure to increasing concentrations of bile acids leads to rapid loss of epithelial barrier function and a consequent increase in mucosal permeability to macromolecules in the Caco-2 cell line (Raimondi et al. 2008). Sustained loss of epithelial barrier function in this way can have severe pathologic implications because it increases the ability of luminal bacteria to penetrate the mucosa, thereby causing inflammation in the human colon (Münch et al. 2007).

The molecular mechanisms by which bile acids alter intestinal epithelial transport still need to be fully clarified. Therefore, bile acids are receiving much attention as significant regulators of mammalian epithelial function in health and disease (Kelly et al., 2013; Hegyi et al. 2018). Crucial differences exist in intestinal tissue structure and function between mammals and fish, and the potential effects of different concentrations of bile salts on intestinal function in fish have received limited attention.

In a recent study, Fuentes et al. (2018) evaluated the epithelial function of physiological (0.4–4 mM) levels of taurocholic and taurolithocholic acids in the intestine of Senegalese sole (*Solea senegalensis*) using the short-circuit current technique. They demonstrated that bile salts alter ionic transport in the intestine, intensifying the absorptive pathway in a dose-dependent and reversible manner without compromising the integrity of the epithelium, at least in vitro*.* The present study was performed bearing in mind the above-detailed effects of bile salts already determined in the sole intestine. We aimed at evaluating the effect of variable concentrations of different bile salts on the epithelial function of the most important aquacultured species in the Mediterranean Basin, the gilthead seabream (*Sparus aurata*), which has been shown to respond positively to bile salt supplementation in growth trials (Ruiz et al. 2023a, 2023b). Considering the growing trend to include bile acid supplements in high-plant diets for fish to compensate for some of their anti-nutritional effects, the study was considered an important preliminary step in decision-making for bile salt type and level of inclusion in feeds for this species.

## Materials and methods

### Chemicals

Bile acids or salts were used in the experiments: a primary bile acid, chenodeoxycholic acid (CDC, Nutriad, Spain); a mixture formed by two primary bile acids, 2.8% cholic acid and 96.7% deoxycholic acid (MIX, Nutriad, Spain); and a conjugated bile salt, sodium taurocholate (TC, Sigma, Madrid). Concentrated stocks (50 mg/ml) were prepared in distilled water to provide final concentrations of 50, 250, and 500 μg/ml in the Ussing chamber during the experiments. Fluorescein isothiocyanate-dextran (FITC mol wt 4000, Sigma) was used as a marker for permeability assays.

### Fish

Sea bream (*Sparus aurata*) juveniles 70–80 g were obtained from the in-house stock of Ramalhete Marine Station (University of Algarve, Faro, Portugal) and maintained in 500-l tanks with running seawater at a density < 5 kg/m^3^ and hand fed three times/week at 1.5% body weight (or the closest they would ingest, considering the low temperature), with a commercial sea bream diet (Aquagold, Sorgal SA, Portugal: 44% crude protein, 14% crude fat, 8% ash, 2.5% crude fibers, 1% phosphorus). Fish were acclimated for 1 month in flowing seawater (salinity 35 p.p.t.; water temperature 16–17 °C) under a natural photoperiod for January in the Algarve, Portugal.

### Tissue removal and definition

In total, tissues from 43 fish were isolated. In most cases, more than one portion of the anterior or the posterior intestine was obtained from each donor fish. In these cases, tissues were allocated to different in vitro experiments, and no tissues from the same intestinal region of an individual fish were included in the same experimental group. The anterior intestine was defined as a portion of about 3 cm in length extending caudally from the point of insertion of the last pyloric caeca and perfectly distinguishable from the mid intestine by the transition of musculature. The posterior intestine was defined as the segment anterior to the rectal sphincter and did not include the rectum, a wider, more flexible region perfectly delimited by the rectal and the anal sphincters. Based on our previous experience of tissue viability (Fuentes et al. 2006), the maximum expected duration of the in vitro experiment was 3.5 h, considering that only a single bile salt type was analyzed in each set of tissues from the same fish in the Ussing chamber.

### Voltage clamp in Ussing chambers

On the day of the experiments, 24-h starved fish were sacrificed, and the whole intestine from stomach to rectum was isolated and transferred to ice-chilled pre-gassed (0.3% CO_2_ + 99.7% O_2_) saline. The intestinal regions defined above were isolated, the luminal content was flushed with saline, the tissue fat was removed, and no attempt was made to remove the serosa muscle layer as previously described (Carvalho et al. 2012). On the day of the experiments, tissues were used in batches of 12 (the maximum number we could run in parallel experiments), mounted on tissue holders of 0.5 cm^2^, and positioned between half-chambers of a P2400 Ussing type chamber (Physiologic Instruments, San Diego, USA) containing 2 ml of physiological saline (160 mmol l^−1^ NaCl, 1 mmol l^−1^ MgSO_4_, 2 mmol l^−1^ NaH_2_PO_4_, 1.5 mmol l^−1^ CaCl_2_, 5 mmol l^−1^ NaHCO_3_, 3 mmol l^−1^ KCl, 5.5 mmol l^−1^ glucose, and 5 mmol l^−1^ HEPES, pH 7.800). During the experiments, the tissue was bilaterally gassed with 0.3% CO_2_ + 99.7% O_2_ and maintained at 17 °C. All experiments were carried out under the short-circuit mode by application of a direct current to set the potential difference (PD) to 0 mV. Under these conditions, changes in short-circuit current, *I*_sc_ (Δ*I*_sc_), represent changes in the transepithelial net ion transfer. Voltage clamping and current injections were performed using VCC MC6 or VCC MC8 voltage clamp amplifiers (Physiologic Instruments, San Diego, USA). Bioelectrical parameters for each tissue were recorded continuously onto LabScribe3 running in a McIntosh computer using an iWorx118 data acquisition system from the time of mounting until the end of the experimental period. *I*_sc_ was continuously recorded, and epithelial resistance (Rt, Ω cm^2^) was manually calculated (Ohm’s law, Rt = Δ*V*/Δ*I*) using the current deflections induced by a bilateral ± 1 mV pulse of 3 s every minute. The apical side of the preparation was considered as the ground. Once Rt and *I*_sc_ achieved a steady state, usually within 25–35 min, control values were recorded, and the preparations received incremental levels of individual bile acids prepared from concentrated stocks at 30-min intervals to provide final concentrations of 50, 250, and 500 μg/ml. Experiments were terminated 30 min after adding the highest concentration of bile salt. Positive values for Δ*I*_sc_ (μAmp/cm^2^) represent stimulation of absorptive currents or inhibition of secretory currents, with the same result regarding net ion movement.

### Permeability assays

In separate experiments, the anterior intestine and the posterior intestine were collected and processed as described above, but no attempt was made to monitor electrophysiological variables. After 30 min of tissue stabilization, the saline was replaced with fresh pre-gassed solution at 17 °C to a final volume of 2 ml per half-chamber. FITC-dextran (average mol wt 4000, Sigma, Madrid) from a concentrated stock (100 mg/ml) was added to achieve a final concentration of 0.5 mg/ml in the apical chamber, and a sample (0.1 ml) was collected from the apical and basolateral compartments after 5 min of mixing to establish time zero. After exactly 1 h, samples from the donor (apical) and receiver (basolateral) compartments were collected into fresh vials and established control periods for apparent permeability (Papp). Individual bile acids from concentrated stocks were sequentially added to the apical chamber to give final concentrations of 250 μg/ml and 500 μg/ml, and permeability was tested in the same tissues for 2 additional hour periods. In these experiments, the 50 μg/ml experimental group was not tested due to putative issues with the preparations’ viability due to the experiments’ length (Fuentes et al. 2006).

Fluorescence measurements were performed using a Multi-Mode Microplate Reader BioTek Synergy™ 4 (BioTek Instruments, Winooski, VT, USA) set for excitation wavelength at 492 nm and emission wavelength at 520 nm. The apparent permeability (Papp) was estimated using the following equation: Papp = (V*dC)/(A*C_0_*dT), where Papp is the permeability in centimeters per second, *V* is the volume of the receiver chamber in μl, *A* is the surface area of the tissue in square centimeters, *C*_0_ is the starting concentration in the donor compartment (apical) in ng/μl, and dC/dT is the rate of concentration change (ng/s) of FITC in the receiving chamber (basolateral*).*

### Statistical analysis

Results are presented as means ± SEM of ten individual tissues per group for the electrophysiology experiments and eight for the permeability measurements. All statistics were performed after assessing the homogeneity of variance (Brown-Forsythe homogeneity test), normality (Kolmogorov–Smirnov normality test), and Rout’s test to identify potential outliers. Comparison of basal parameters between intestinal regions was carried out by the Student *t*-test. Effects of bile salts were evaluated with a two-way analysis of variance, considering the intestinal region and bile salt dose as main factors, complemented with the Sidak test to identify significant effects. All statistical analyses were performed with Prism 8.0 (GraphPad Prism 8.0 for McIntosh, GraphPad Software, San Diego, California, USA), and groups were considered significantly different at *p* < 0.05.

Multivariate analysis was performed using the software Primer v7.0.21 from PRIMER-e (Massey University, Albany, New Zealand; Clarke and Gorley 2015). For each parameter under study (delta current, resistance, and permeability), the corresponding resemblance matrix (Euclidean distance) was obtained using all samples or samples from the same biliary salt experiment. The following routines were then used in sequence: (i) CLUSTER to obtain hierarchical clustering into sample groups and the respective cophenetic correlation, together with SIMPROF to test for structure in the data and identify sample groups with 95% confidence, and (ii) permutation-based hypothesis testing (ANOSIM), an analog of univariate ANOVA, run on the resemblance matrix to test for differences between groups of samples from different experimental treatments (different biliary salts, same intestinal region) and between groups of samples from the same treatment (same biliary salt, two intestinal regions). Finally, a principal coordinate (PCO) analysis was used to produce a configuration plot to visualize the level of similarity of individual samples of each dataset.

## Results

### Basal measurements in sea bream intestine

Basal epithelial parameters of intestinal preparations in the sea bream anterior and posterior intestine are shown in Table 1. In symmetric conditions and under voltage clamp, the intestine of sea bream shows significantly different short-circuit currents (*I*_sc_) between the anterior and the posterior intestine. In addition, tissue resistance (Rt) was significantly higher in the anterior than in the posterior intestine, showing 166.6 ± 15.9 vs. 78.1 ± 3.9 Ω cm^2^, respectively. Apparent permeability measured using 4 kD FITC-dextran was between 2 and 3 × 10^−7^ cm/s in both regions, without significant differences.

**Table 1:** Basal epithelial parameters of short-circuit current (*I*_sc_, μAmp/cm^2^), tissue resistance (Rt, Ω cm^2^), and permeability (Papp, × 10^−7^ cm/s) in the anterior and posterior intestine of the sea bream (*Sparus aurata*) used in this experiment. The number of tissues for each parameter is given in parentheses. Asterisks represent differences between the anterior and posterior intestine (Student *t*-test, *p* < 0.001)

	Anterior Intestine	Posterior Intestine
*I*_sc_ (μAmp/cm^2^)	1.13 ± 0.28 (30)	3.94 ± 0.49 (30)*
Rt (Ω cm^2^)	166.6 ± 15.9 (30)	78.1 ± 3.9 (30)*
Papp (× 10^−7^ cm/s)	2.83 ± 0.49 (24)	2.03 ± 0.42 (24)

### Effects of bile acids on short-circuit current

**Fig. 1:**
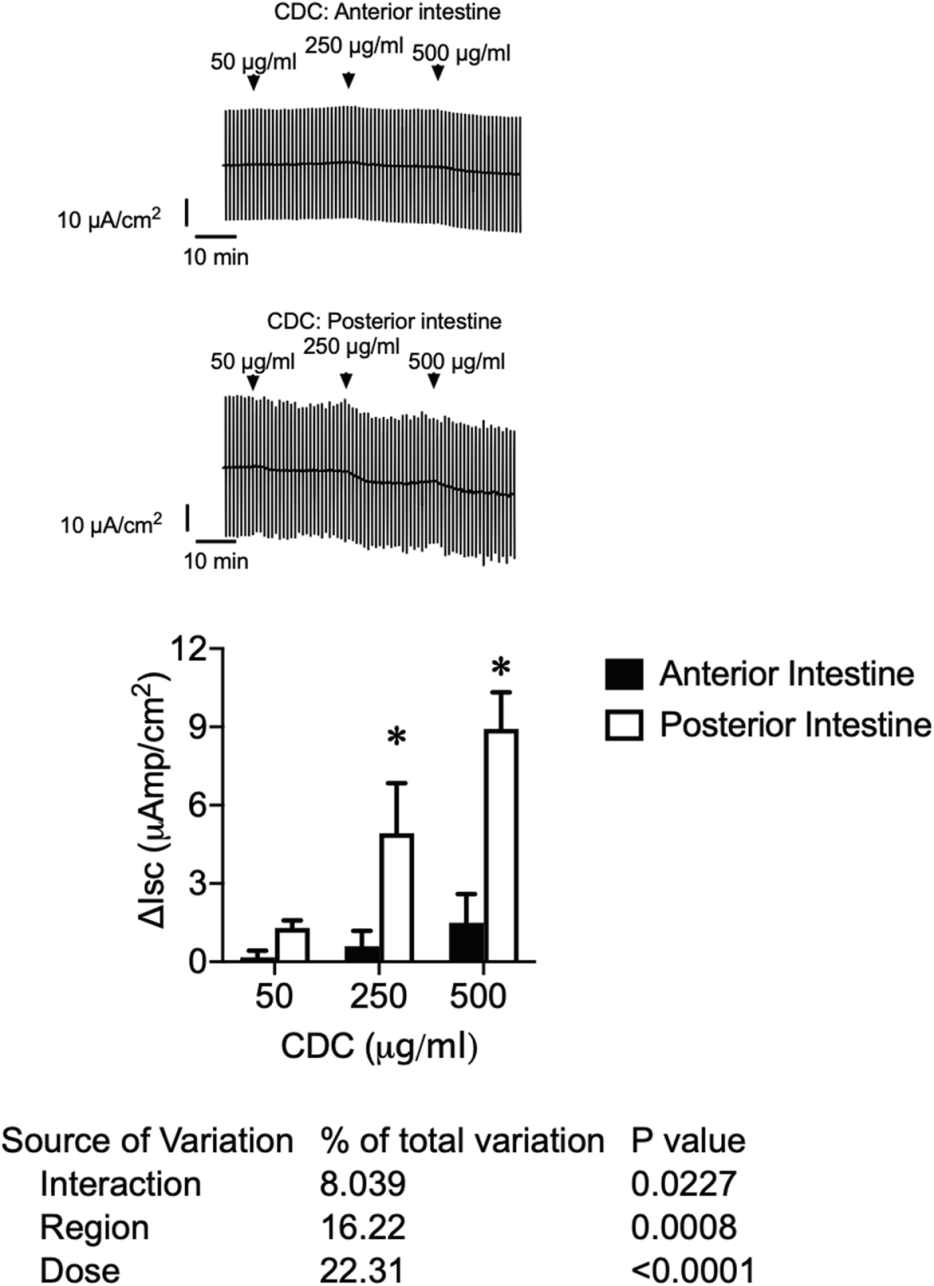
Original traces of short-circuit current (*I*_sc_, μAmp/cm^2^) in the anterior intestine and the posterior intestine of sea bream juveniles were recorded after consecutive apical application of CDC 50, 250, and 500 μg/ml. Vertical current deflections are generated by 1 mV pulses to calculate Rt. Lower panel: changes in short-circuit current (Δ*I*_sc_, μAmp/cm.^2^) in sea bream juveniles’ anterior and posterior intestines after consecutive apical application of CDC 50, 250, and 500 μg/ml. Each column represents the average + SEM (*n* = 10). Asterisks represent significant differences between regions for a given dose (two-way ANOVA, followed by Sidak multi-comparison test)

**Fig. 2:**
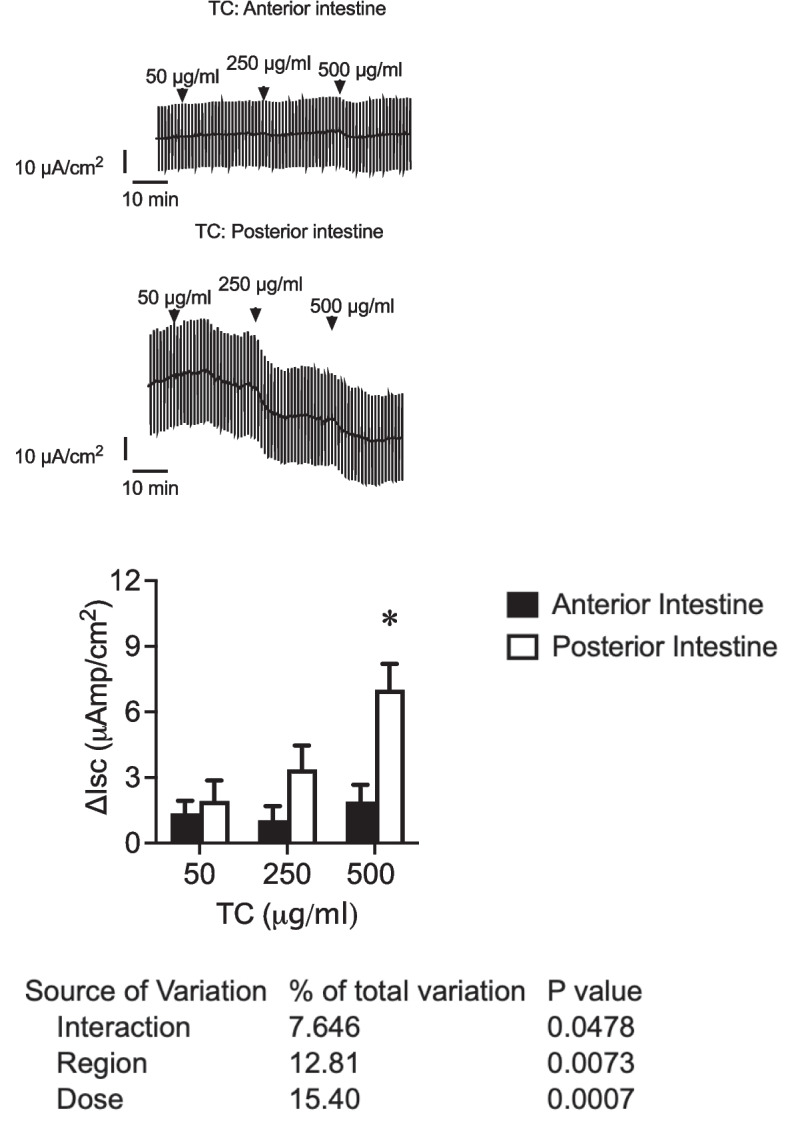
Original traces of short-circuit current (*I*_sc_, μAmp/cm^2^) in sea bream juveniles’ anterior intestine and posterior intestine were recorded after consecutive apical application of TC 50, 250, and 500 μg/ml. Vertical current deflections are generated by 1 mV pulses to calculate Rt. Lower panel: changes in short-circuit current (Δ*I*_sc_, μAmp/cm.^2^) in the anterior and posterior intestine of sea bream juveniles after consecutive apical application of TC 50, 250, and 500 μg/ml. Each column represents the average + SEM (*n* = 10). Asterisks represent significant differences between regions for a given dose (two-way ANOVA, followed by Sidak multi-comparison test)

**Fig. 3:**
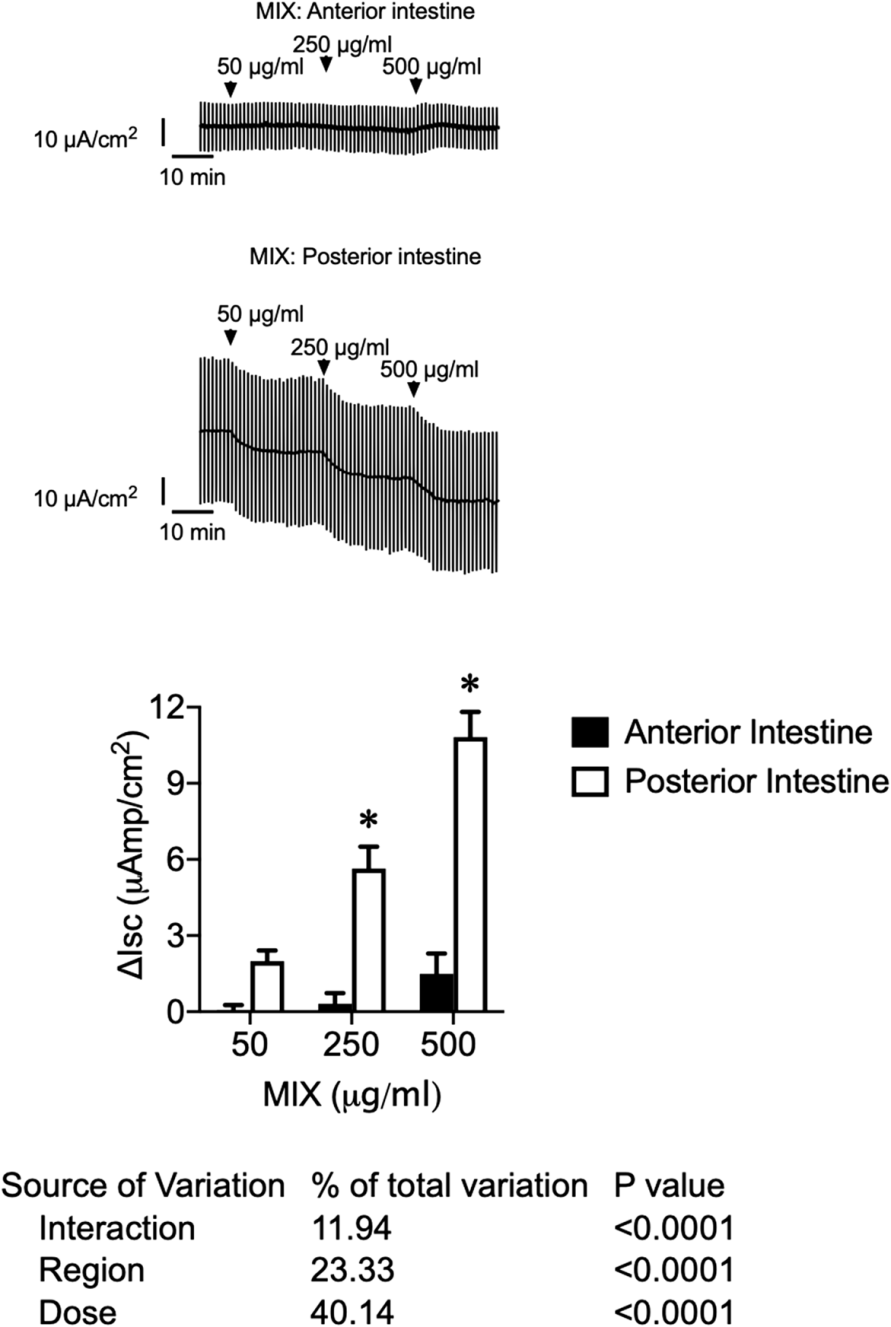
Original traces of short-circuit current (*I*_sc_, μAmp/cm^2^) in the anterior intestine and the posterior intestine of sea bream juveniles were recorded after consecutive apical application of MIX 50, 250, and 500 μg/ml. Vertical current deflections are generated by 1 mV pulses to calculate Rt. Lower panel: changes in short-circuit current (Δ*I*_sc_, μAmp/cm.^2^) in the anterior and posterior intestine of sea bream juveniles after consecutive apical application of MIX 50, 250, and 500 μg/ml. Each column represents the average + SEM (*n* = 10). Asterisks represent significant differences between regions for a given dose (two-way ANOVA, followed by Sidak multi-comparison test)

Increasing CDC concentrations, 50, 250, and 500 μg/ml applied in Ussing chambers, did not produce an effect in the anterior intestine Δ*I*_sc_ (μAmp/cm^2^). However, the posterior intestine responded with graded increases of Δ*I*_sc_ in a dose-dependent manner. The two-way ANOVA analysis (Fig. 1) evidenced significant effects of the dosage (*p* < 0.001) and the intestinal region (*p* = 0.008), as well as a significant interaction between both factors (*p* = 0.0227). Similar results were obtained when testing increasing concentrations of TC and the bile acid MIX (Figs. 2 and 3).

### Effects of bile acids on tissue resistance

**Fig. 4:**
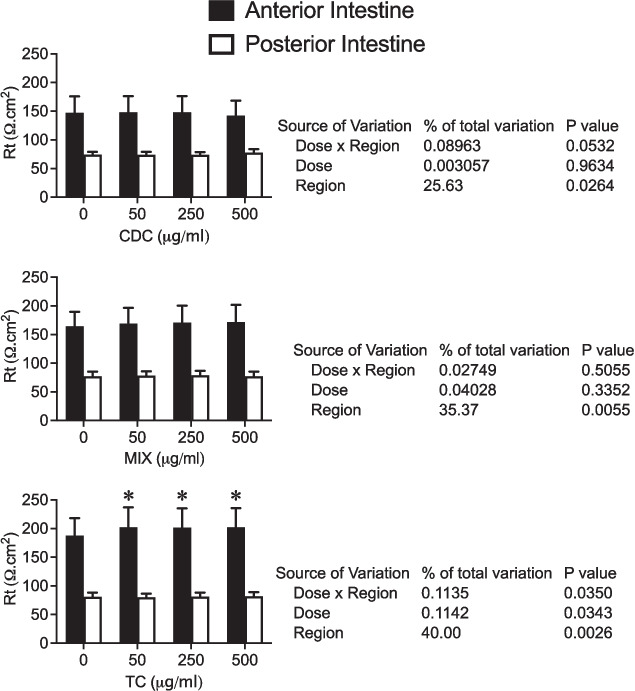
Tissue resistance (Rt, Ω cm.^2^) of juvenile sea bream anterior and posterior intestine in basal conditions and in response to consecutive apical application of 50, 250, and 500 μg/ml of CDC, MIX, and T009 (upper, central, and lower panel, respectively). Each column represents the average + SEM (*n* = 10). Asterisks represent significant differences from corresponding basal values (two-way ANOVA, followed by Sidak multi-comparison test)

Increasing concentrations of CDC and MIX (50, 250, and 500 μg/ml) applied to in vitro preparations in Ussing chambers did not affect tissue resistance. However, applying TC significantly increased tissue resistance in the anterior but not in the posterior intestine. Results from the two-way ANOVA (Fig. 4) showed a significant effect of the dosage (*p* = 0.0343) and the intestinal region (0.0026), as well as a significant interaction (*p* = 0.035) between both factors.

### Effects of bile acids on tissue permeability

**Fig. 5:**
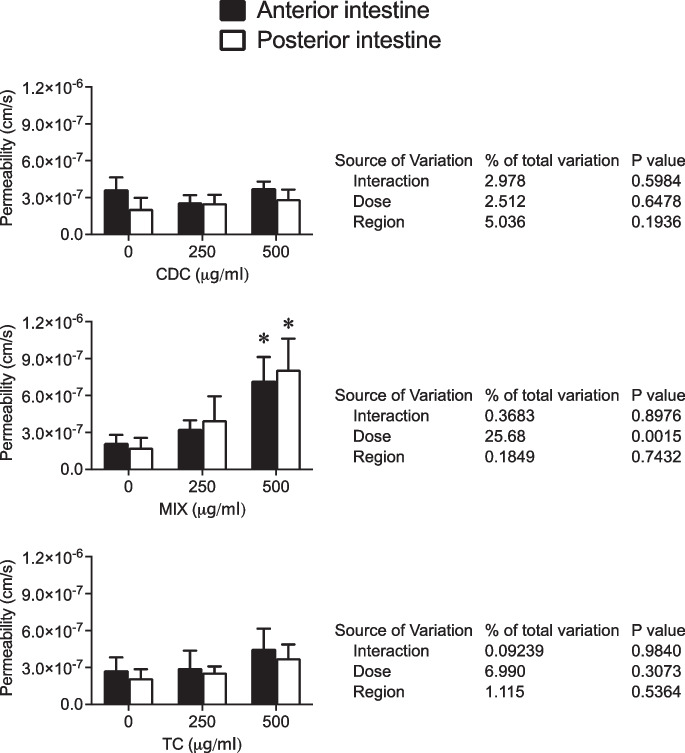
Permeability (Papp, cm/s) of juvenile sea bream anterior and posterior intestine in basal conditions and in response to consecutive apical application of 250 and 500 μg/ml of CDC, MIX, and T009 (upper, central, and lower panel, respectively). Each column represents the average + SEM (*n* = 8). Asterisks represent significant differences from corresponding basal values (two-way ANOVA, followed by Sidak multi-comparison test)

Figure 5 shows the effects of CDC, MIX, and TC on tissue permeability in the anterior and posterior sea bream intestine. The application of increasing concentrations of CDC and TC (250 and 500 μg/ml) produced no effect either in the anterior or posterior intestine (*p* > 0.05, two-way ANOVA). However, increasing doses of bile acid MIX determined a significant permeability increase (*p* = 0.0015), without effects at levels of 250 μg/ml (two-way ANOVA followed by Sidak multi-comparison test), regardless of the intestinal region (Fig. 5).

### Multivariate analysis

The multivariate analysis performed considering either of the three parameters under study showed neither significant differences between the overall effect of CDC, MIX, or TC nor between the respective effect of each biliary salt in the anterior or the posterior intestinal regions. However, significant differences were detected between the two intestinal areas for the effect of CDC in the delta current (Supplementary Fig. 1; ANOSIM (*R*) = 0.849; *p* = 0.001) and in the permeability (Supplementary Fig. 2; ANOSIM (*R*) = 0.835; *p* = 0.003) and for the effect of MIX in delta current (Supplementary Fig. 2; ANOSIM (*R*) = 0.987; *p* = 0.001). A borderline difference was detected (ANOSIM (*R*) = 0.51; *p* = 0.002) between the effect of TC on the epithelial resistance of each intestinal region.

## Discussion

The primary objective of this study was to unveil the physiological basis that sustains the potential impact of bile salts on the epithelial functionality of the sea bream’s intestine. We approached the work taking into consideration specific bile salts, the relative concentration, and the specific intestinal region being investigated. To accomplish this, we utilized the Ussing chamber, a specific methodology that allows for the measurement of three different parameters: the short-circuit current, tissue resistance, and permeability. The short-circuit current is an indicator of the net active transcellular ion transport across the intestinal epithelium. The process of active ion transport results in the establishment of a potential difference across the epithelium, referred to as TEP (transepithelial potential). We measured this voltage difference using two electrodes positioned as closely as possible to the tissue or epithelium. To eliminate any spontaneous voltage, we introduced a counter-current using another set of two current electrodes at a distance from the epithelium. This externally applied current is known as the short-circuit current (*I*_sc_) and provides an exact measurement of net ion transport across the epithelium when the tissue is short-circuited; *I*_sc_ serves as a precise reflection of the tissue’s absorptive or secretory capacity (Clarke 2009). On the other hand, barrier functionality is primarily governed by epithelial cells and tight junctions, and in the context of epithelial systems, tissue resistance is regarded as the electrical manifestation of barrier function (Clarke 2009) and the bioelectrical evaluation of resistance/integrity of the tissue. Preserving the integrity of the barrier function within the intestinal epithelium is of utmost importance as it segregates the internal and external compartments of the body (Clarke 2009). Numerous factors can potentially influence intestinal permeability, including alterations in gut microbiota, mucus layer changes, and epithelial lining damage, that can lead to translocation and infiltration of luminal contents to the intestinal tissue and plasma (Gieryńska et al. 2022; Stolfi et al. 2022). In the context of the fish intestine, this approach has been employed to assess insults to the epithelia resulting from the introduction of plant protein or alternative protein sources in sea bream (Estensoro et al. 2016; Aragão et al. 2020; Molina-Roque et al. 2022), sea bass (Fonseca et al. 2023) or meagre (Sáenz de Rodrigáñez et al. 2013).

In the present study, we tested the putative effects of selected bile salts chenodeoxycholic acid (CDC), a mixture formed by two bile acids, 3% cholic acid and 97% deoxycholic acid (MIX), and a conjugated bile salt sodium taurocholate (TC) on the ion transport in the intestine of sea bream. The selection of the CDC and the mixture used in this study was based on previous research concerning the bile composition in fish, detailed in the “Introduction” section. For instance, chenodeoxycholic acid (CDC) was identified in the grass carp *Ctenopharyngodon idella*, as reported by Zhang et al. (2017). In contrast, aside from the Cypriniformes, primarily characterized by C27 bile alcohols (Hagey et al. 2010), most cultivated fish species are characterized by the prevalence of C24 bile acids, and, in the case of Sparids like the red seabream (*Pagrosomus major*), CDC may account by 40% of total bile acids (Goto et al 1996).

Notably, these bile acids are primarily conjugated with taurine, with taurine being the sole amino acid involved in the conjugation of bile acids in these species (Kortner et al. 2016; Kim et al. 2015; Yamamoto et al. 2007). The levels tested in vitro in this study were selected with the assumption of feeding at approximately 1.5% of the body weight per day, enriched with 50–500 mg bile acids per kg of feed. These values correspond to intermediate supplementation ranges identified in the literature for fish (Kim et al. 2015; Hagey et al. 2010; Goto et al. 1996). For a fish weighing around 100 g and considering a feeding rate of 1.5%, the ingestion rate of bile acids at 50–500 mg/kg of feed would amount to an estimated intake of 75–750 μg per fish, a range aligning with the most commonly published values (see above). These bile acids are anticipated to be retained in the intestinal lumen, resulting in concentrations ranging from 50 to 500 μg/ml in the intestinal fluid, the range we used in our in vitro experiments. While some intestinal fluid may be lost through absorption into the bloodstream, there is also the potential for the uptake of bile salts in the anterior intestine, which could decrease luminal levels. However, if water absorption occurs throughout the intestine as it does along the intestine of the sea bream (Carvalho et al. 2012), both regions might maintain comparable concentrations.

We applied bile salts always on the apical/luminal compartment of our in vitro assays to simulate the putative effects of feed-bound bile salts in the intestine of sea bream. Results evidenced that the addition of either CDC or MIX at increasing concentrations did not affect tissue resistance either in the anterior or posterior intestine. In contrast, sodium taurocholate induced a slight yet significant increase in tissue resistance in the anterior intestine of the sea bream. This observation was surprising since in our previous study, performed on the intestine of another fish species, the Senegalese sole, neither taurocholic nor taurolithocholic acids affected tissue resistance (Fuentes et al. 2018). However, sodium taurocholate decreases tissue resistance in other epithelial models, such as the rabbit gastric mucosa (Birkett and Silen 1974), although at much higher concentrations (2.5 to 20 mM). These observations suggest that the effects on barrier function are both bile salt and species-specific, possibly exhibiting heterogeneity, as different sections of the intestine may respond differently. This is a crucial aspect to take into account when considering the potential utilization of a specific bile salt as a dietary supplement.

Electrophysiological measurements represent the gold standard for assessing tissue integrity. Nevertheless, we further characterized the barrier function concerning the influence of bile salts using the fluorescent small-size permeability marker FITC-dextran, which has a molecular weight of 4 kD. Our observations revealed no significant impact on permeability in tissues stimulated with either CDC or taurocholate. However, when tissues were stimulated with the MIX, we observed increased permeability at the highest concentration employed (500 μg/ml), which roughly equates to a range of 1–1.5 mM in the luminal compartment. This finding suggests that while the MIX may display favorable effects on the transcellular absorption pathway, as evidenced by the stimulation of *I*_sc_, it could have adverse effects at higher concentrations, potentially leading to a disruption in intestinal barrier function. Similar effects have been observed in other models, such as the Caco-2 cell intestinal model (Zeng et al. 2022). However, whether the effect described here is temporary and reversible or results in a lasting disruption of the intestinal barrier function is a subject that requires further investigation. In addition, we need to consider how our results in vitro translate to an in vivo situation. Bile salt metabolism is complex and can be regulated by various factors, for example, bile acid synthesis autoregulation through the activation of the farnesoid X receptor (FXR) in the liver and intestine, which causes hepatic cyp7a1 not to be activated and a reduced bile acid synthesis (Romano et al. 2020).

When examining together the present findings made on seabream in combination with those previously obtained with the Senegalese sole in vitro (Fuentes et al. 2018), a clear pattern emerges, pointing to more pronounced effects of bile salts on the ion transport in the posterior regions of the fish intestine. While our analysis focused on the impact of bile salts in distinct segments of the seabream’s intestinal tract, specifically the most anterior and posterior sections, results reveal a progressively increasing influence on the absorption process from the anterior to the posterior regions of the intestine. Multivariate analysis revealed distinct responses between intestinal regions to each bile salt, albeit with variations in the affected parameters. Specifically, with CDC, disparities were evident in both delta current and permeability responses. In contrast, with MIX, discrepancies were solely detected in delta current, and with TC, alterations were observed exclusively in epithelial resistance. However, the extent of significance attributed to the region-specific effects of bile salts warrants further investigation, as molecular mechanisms influenced by the chemical conformation of the bile salts utilized cannot be discounted.

In conclusion, the present study explored the effects of different bile salts on the ion transport in the intestine of sea bream. All bile salts had some stimulatory effects in the absorptive pathway, apparent region-dependent effects, and concentration-dependent stimulation. In addition, the mix of cholic and deoxycholic acids suggested a potential disruption of the intestinal barrier function. Although the degree of relevance associated with the region-dependent function of bile salts on ion transport remains a subject for further exploration, the effects described here are probably complexly linked to nutrient absorption. The uptake of protein components, encompassing amino acids and small peptides, demands a coordinated interplay between nutrient and ion transport mechanisms. This interplay connects nutrient sensing with the absorption of nutrients through the regulation of ion transport, a process mediated by enteroendocrine cells (McCauley et al., 2020). Given that a substantial portion of dietary amino acid and peptide transport relies on sodium (Na^+^)-and hydrogen (H^+^)-linked transporters (Broer and Fairweather 2018; Broer 2008), precise adjustments of ion concentrations within the intestinal lumen become paramount for maintaining and potentially enhancing the absorptive processes crucial for proper nutrition. Bile salts, whether acting individually or as a collective pool, are likely pivotal in coordinating nutrient absorption by influencing the function of epithelial ion transport. Considering this, using bile salts as feed additives could be contemplated mostly in fish diets, including ingredients whose composition may impair the normal intestinal absorption process.

### Supplementary Information

Below is the link to the electronic supplementary material.

Supplementary file1 (DOCX 222 KB)

## Data Availability

Data is provided within the manuscript. Derived data supporting the findings of this study are available from the corresponding author L. Fuentes on request.

## References

[CR1] Aragão C, Colen R, Ferreira S, Pinto W, Conceição LEC, Dias J (2014) Microencapsulation of taurine in Senegalese sole diets improves its metabolic availability. Aquaculture 431:53–58 10.1016/j.aquaculture.2014.04.041

[CR2] Aragão C, Cabano M, Colen R, Fuentes J, Dias J (2020) Alternative formulations for gilthead seabream diets: towards a more sustainable production. Aquac Nutr 26:444–455. 10.1111/anu.13007 10.1111/anu.13007

[CR3] Birkett D, Silen W (1974) Alteration of the physical pathways through the gastric mucosa by sodium taurocholate. Gastroenterology 67(6):1131–1138 4430426 10.1016/S0016-5085(19)32698-8

[CR4] Broer S (2008) Amino acid transport across mammalian intestinal and renal epithelia. Physiol Rev 88:249–286. 10.1152/physrev.00018.2006 18195088 10.1152/physrev.00018.2006

[CR5] Broer S, Fairweather SJ (2018) Amino acid transport across the mammalian intestine. Comp Physiol 9:343–373. 10.1002/cphy.c170041 10.1002/cphy.c170041 30549024

[CR6] Carvalho ES, Gregorio SF, Power DM, Canario AVM, Fuentes J (2012) Water absorption and bicarbonate secretion in the intestine of the sea bream: heterogeneous response to transmembrane and soluble adenylyl cyclase stimulation. J Comp Physiol B 182(8):1069–1080 22752677 10.1007/s00360-012-0685-4

[CR7] Chesney RW, Helms RA, Christensen M, Budreau AM, Han X, Sturman JA (1998) The role of taurine in infant nutrition. Adv Exp Med Biol 442:463–476 9635063 10.1007/978-1-4899-0117-0_56

[CR8] Chiang JY (2009) Bile acids: regulation of synthesis. J Lipid Res 50(10):1955–1966. 10.1194/jlr.R900010-JLR200 19346330 10.1194/jlr.R900010-JLR200 PMC2739756

[CR9] Clarke LL (2009) A guide to Ussing chamber studies of mouse intestine. Am J Physiol Gastrointest Liver Physiol 296:G1151–G1166. 10.1152/ajpgi.90649.2008 19342508 10.1152/ajpgi.90649.2008 PMC2697950

[CR10] Clarke KR, Gorley RN (2015) PRIMER v7: user manual/tutorial, 3rd edn. Plymouth, United Kingdom: Primer-E Ltd

[CR11] Estensoro I, Ballester-Lozano G, Benedito-Palos L, Grammes F, Martos-Sitcha JA, Mydland L-T, Calduch-Giner JA, Fuentes J, Karalazos V, Ortiz A, Øverland M, Sitjà-Bobadilla A, Pérez-Sánchez J (2016) Dietary butyrate helps to restore the intestinal status of a marine teleost (*Sparus**aurata*) fed extreme diets low in fish meal and fish oil. PlosONE 11(11):e0166564. 10.1371/journal.pone.0166564 10.1371/journal.pone.0166564 PMC5127657 27898676

[CR12] Fonseca F, Fuentes J, Vizcaíno AJ, Alarcón FJ, Mancera JM, Martínez-Rodríguez G, Martos-Sitcha JS (2023) From invasion to fish fodder: inclusion of the brown algae Rugulopteryx okamurae in aquafeeds for European sea bass Dicentrarchus labrax (L., 1758). Aquaculture 568:739318 10.1016/j.aquaculture.2023.739318

[CR13] Francis G, Makkar HPS, Becker K (2001) Antinutritional factors present in plant-derived alternate fish feed ingredients and their effects in fish. Aquaculture 199:197–227 10.1016/S0044-8486(01)00526-9

[CR14] Fuentes J, Figueiredo J, Power DM, Canario AVM (2006) Parathyroid hormone-related protein regulates intestinal calcium transport in the sea bream (*Sparus**auratus*). Am J Physiol Regul Integr Comp Physiol 291:R1499–R150 16763076 10.1152/ajpregu.00892.2005

[CR15] Fuentes J, Ribeiro L, Aragão C (2018) Bile salts regulate ion transport in the intestine of Senegalese sole. Aquaculture 495:842–848. 10.1016/j.aquaculture.2018.06.050 10.1016/j.aquaculture.2018.06.050

[CR16] Giaquinto PC, Hara TJ (2008) Discrimination of bile acids by the rainbow trout olfactory system: evidence as potential pheromone. Biol Res 41:33–42. 10.4067/S0716-97602008000100005 18769761 10.4067/S0716-97602008000100005

[CR17] Gieryńska M, Szulc-Dąbrowska L, Struzik J, Mielcarska MB, Gregorczyk-Zboroch KP (2022Jan 8) Integrity of the intestinal barrier: the involvement of epithelial cells and microbiota-a mutual relationship. Animals (basel) 12(2):145. 10.3390/ani12020145 35049768 10.3390/ani12020145 PMC8772550

[CR18] Goto T, Une T, Kihira K, Kuramoto T, Hoshita T (1993) Enzymatic formation of D-cysteinolic acid conjugated chanodeoxycolic acid in liver preparation from red seabream. Pagrosomus Major Biol Pharm Bull 16(12):1216–1219 8130769 10.1248/bpb.16.1216

[CR19] Goto T, Ui T, Une T, Kuramoto T, Kirira T, Hoshita T (1996) Bile salt composition and distribution of the D-cystenolic acid conjugated bile salts in fish. Fish Sci 62:606–609 10.2331/fishsci.62.606

[CR20] Gu M, Bai N, Zhang YQ, Krogdahl Å (2016) Soybean meal induces enteritis in turbot Scophthalmus maximus at high supplementation levels. Aquaculture 464:286–95. 10.1016/j.aquaculture.2016.06.035 10.1016/j.aquaculture.2016.06.035

[CR21] Hagey LR, Møller PR, Hofmann AF, Krasowski MD (2010) Diversity of bile salts in fish and amphibians: evolution of a complex biochemical pathway. Physiol Biochem Zool 83(2):308–321. 10.1086/649966 20113173 10.1086/649966 PMC2845723

[CR22] Hegyi P, Maléth J, Walters JR, Hofmann AF, Keely SJ (2018) Guts and gall: bile acids in regulation of intestinal epithelial function in health and disease. Physiol Rev 98(4):1983–2023. 10.1152/physrev.00054.2017 30067158 10.1152/physrev.00054.2017

[CR23] Hofmann AF, Hagey LR, Krasowski MD (2010) Bile salts of vertebrates: structural variation and possible evolutionary significance. J Lipid Res 51(2):226–246. 10.1194/jlr.R000042 19638645 10.1194/jlr.R000042 PMC2803226

[CR24] Iwashita Y, Suzuki N, Yamamoto T, Shibata J, Isokawa K, Soon AH, Ikehata Y, Furuita H, Sugita T, Goto T (2007) Supplemental effect of cholyltaurine and soybean lecithin to a soybean meal-based fish meal-free diet on hepatic and intestinal morphology of rainbow trout *Oncorhynchus mykiss*. Fish Sci 74:1083–1095. 10.1111/j.1444-2906.2008.01628.x.] 10.1111/j.1444-2906.2008.01628.x.]

[CR25] Iwashita Y, Suzuki N, Matsunari H, Sugita T, Yamamoto T (2008) Influence of soya saponin, soya lectin, and cholyltaurine supplemented to a casein-based semipurified diet on intestinal morphology and biliary bile status in fingerling rainbow trout *Oncorhynchus mykiss*. Fish Sci 75:1307–1315. 10.1007/s12562-009-0158-1 10.1007/s12562-009-0158-1

[CR26] Jin M, Pan T, Cheng X, Zhu TT, Sun P, Zhou F, Ding X, Zhou Q (2019) Effects of supplemental dietary L-carnitine and bile acids on growth performance, antioxidant and immune ability, histopathological changes and inflammatory response in juvenile black seabream (*Acanthopagrus schlegelii*) fed high-fat diet. Aquaculture 504:199–209. 10.1016/j.aquaculture.2019.01.063 10.1016/j.aquaculture.2019.01.063

[CR27] Katona BW, Anant S, Covey DF, Stenson WF (2009) Characterization of enantiomeric bile acid-induced apoptosis in colon cancer cell lines. J Biol Chem 284(5):3354–3364. 10.1074/jbc.M805804200 19054763 10.1074/jbc.M805804200 PMC2631943

[CR28] Kelly OB, Mroz MS, Ward JB, Colliva C, Scharl M, Pellicciari R, Gilmer JF, Fallon PG, Hofmann AF, Roda A, Murray FE, Keely SJ (2013) Ursodeoxycholic acid attenuates colonic epithelial secretory function. J Physiol 591(2307–2318):2013. 10.1113/jphysiol.2013.252544 10.1113/jphysiol.2013.252544 PMC3650696 23507881

[CR29] Kim S-K, Kim K-G, Kim K-D, Kim K-W, Son M-H, Rust M, Johnson R (2015) Effect of dietary taurine levels on the conjugated bile acid composition and growth of juvenile Korean rockfish *Sebastes schlegeli* (Hilgendorf). Aquac Res 46:2768–2775 10.1111/are.12431

[CR30] Kortner TM, Bjorkhem I, Krasnov A, Timmerhaus G, Krogdahl A (2014) Dietary cholesterol supplementation to a plant-based diet suppresses the complete pathway of cholesterol synthesis and induces bile acid production in Atlantic salmon (Salmo salar L.). Br J Nutr 111:2089–103. 10.1017/S0007114514000373 24635969 10.1017/S0007114514000373

[CR31] Kortner TM, Penn MH, Bjӧrkhem I, Måsøval K, Krogdahl Å (2016) Bile components and lecithin supplemented to plant based diets do not diminish diet related intestinal inflammation in Atlantic salmon. BMC Vet Res 12(1):190. 10.1186/s12917-016-0819-0 27604133 10.1186/s12917-016-0819-0 PMC5015236

[CR32] Krogdahl Å, Penn M, Thorsen J, Refstie S, Bakke AM (2010) Important antinutrients in plant feedstuffs for aquaculture: an update on recent findings regarding responses in salmonids. Aquacult Res 41:333–344 10.1111/j.1365-2109.2009.02426.x

[CR33] McCauley HA, Matthis AL, Enriquez JR, Nichol JT, Sanchez JG, Stone WJ, Sundaram N, Helmrath MA, Montrose MH, Aihara E, Wells JM (2020) Enteroendocrine cells couple nutrient sensing to nutrient absorption by regulating ion transport. Nature Communications 11(1). 10.1038/s41467-020-18536-z 10.1038/s41467-020-18536-z PMC7508945 32963229

[CR34] Molina-Roque L, Bárany A, Sáez MI, Alarcón FJ, Tapia ST, Fuentes J, Mancera JM, Perera E, Martos-Sitcha JA (2022) Biotechnological treatment of microalgae enhances growth performance, hepatic carbohydrate metabolism and intestinal physiology in gilthead seabream (*Sparus aurata*) juveniles close to commercial size. Aquaculture Reports 25:101248 10.1016/j.aqrep.2022.101248

[CR35] Münch A, Ström M, Söderholm JD (2007) Dihydroxy bile acids increase mucosal permeability and bacterial uptake in human colon biopsies. Scand J Gastroenterol 42(10):1167–1174. 10.1080/00365520701320463 17852874 10.1080/00365520701320463

[CR36] Raimondi F, Santoro P, Barone MV, Pappacoda S, Barretta ML, Nanayakkara M, Apicella C, Capasso L, Paludetto R (2008) Bile acids modulate tight junction structure and barrier function of Caco-2 monolayers via EGFR activation. Am J Physiol Gastrointest Liver Physiol 29:G906–G913. 10.1152/ajpgi.00043.2007 10.1152/ajpgi.00043.2007 18239063

[CR37] Rolen SH, Caprio J (2008) Bile salts are effective taste stimuli in channel catfish. J Exp Biol 21:2786–2791. 10.1242/jeb.018648 10.1242/jeb.018648 18723536

[CR38] Romano N, Kumar V, Yang G, Kaibaf K, Rubio M, Overturf KE, Brezas A, Hardy R (2020) Bile acid metabolism in fish: disturbances caused by fishmeal alternatives and some mitigating effects from dietary bile inclusions. Aquaculture 12(3):1792–1817. 10.1111/raq.12410 10.1111/raq.12410

[CR39] Ruiz A, Andree KB, Sanahuja I, Holhorea PG, Calduch-Gine JA, Morais S, Pastor JJ, Pérez-Sánchez J, Gisbert E (2023a) Bile salt dietary supplementation promotes growth and reduces body adiposity in gilthead seabream (*Sparus**aurata*). Aquaculture 566:739203. 10.1016/j.aquaculture.2022.739203 10.1016/j.aquaculture.2022.739203

[CR40] Ruiz A, Andree KB, Furones D, Holhorea PG, Calduch-Giner JÀ, Viñas M, Pérez-Sánchez J, Gisbert E (2023) Modulation of gut microbiota and intestinal immune response in gilthead seabream (*Sparus**aurata*) by dietary bile salt supplementation. Front Microbiol 14:1123716. 10.3389/fmicb.2023.1123716 37168118 10.3389/fmicb.2023.1123716 PMC10166234

[CR41] Sáenz de Rodrigáñez MA, Fuentes J, Moyano FJ, Ribeiro L (2013) *In vitro* evaluation of the effect of a high plant protein diet and nucleotide supplementation on intestinal integrity in meagre (*Argyrosomus regius*). Fish Physiol Biochem 39:1365–1370 23525861 10.1007/s10695-013-9790-x

[CR42] Staessen TWO, Verdegem MCJ, Koletsi P, Schrama JW (2020) The effect of dietary protein source (fishmeal vs. plant protein) and non-starch polysaccharide level on fat digestibility and faecal bile acid loss in rainbow trout (Oncorhynchus mykiss). Aquac Res 51(3):1170–1181. 10.1111/are.14467 10.1111/are.14467

[CR43] Stolfi C, Maresca C, Monteleone G, Laudisi F (2022) Implication of intestinal barrier dysfunction in gut dysbiosis and diseases. Biomedicines 10(2):289. 10.3390/biomedicines10020289 35203499 10.3390/biomedicines10020289 PMC8869546

[CR44] Tocher DR, Bendiksen EA, Campbell PJ, Bell JG (2008) The role of phospholipids in nutrition and metabolism of teleost fish. Aquaculture 280:21–34 10.1016/j.aquaculture.2008.04.034

[CR45] Yamamoto T, Suzuki N, Furuita H, Sugita T, Tanaka N, Goto T (2007) Supplemental effect of bile salts to soybean meal-based diet on growth and feed utilization of rainbow trout *Oncorhynchus mykiss*. Fish Sci 73:123–131. 10.1111/j.1444-2906.2007.01310.x 10.1111/j.1444-2906.2007.01310.x

[CR46] Yui S, Kanamoto R, Saeki T (2009) Biphasic regulation of cell death and survival by hydrophobic bile acids in HCT116 cells. Nutr Cancer 61(3):374–380. 10.1080/01635580802582744 19373611 10.1080/01635580802582744

[CR47] Zeng Huawei, Safratowich Bryan D, Cheng Wen-Hsing, Larson Kate J, Briske-Anderson Mary (2022) Deoxycholic acid modulates cell-junction gene expression and increases intestinal barrier dysfunction. Molecules 27(3):723. 10.3390/molecules27030723 35163990 10.3390/molecules27030723 PMC8839472

[CR48] Zhang C, Brown SB, Hara TJ (2001) Biochemical and physiological evidence that bile acids produced and released by lake char (*Salvelinus**namaycush*) function as chemical signals. J Comp Physiol B 171:161–171 11302533 10.1007/s003600000170

[CR49] Zhang J, Xiong F, Wang GT, Li WX, Li M, Zou H, Wu SG (2017) The influence of diet on the grass carp intestinal microbiotaand bile acids. Aquaculture Research 48(9):4934–4944. 10.1111/are.13312 10.1111/are.13312

