# Supplementary material for: Effect of bile salts on intestinal epithelial function in gilthead seabream (Sparus aurata)
Source: Fish Physiol Biochem. 2024 Jun 25;50(4):1777–90. doi: 10.1007/s10695-024-01369-8 (PMC11286703; doi:10.1007/s10695-024-01369-8)
Supplement: Supplementary file 1 — Supplementary file1 (DOCX 222 KB) [file 10695_2024_1369_MOESM1_ESM.docx]

**SUPLEMENTARY MATERIAL**


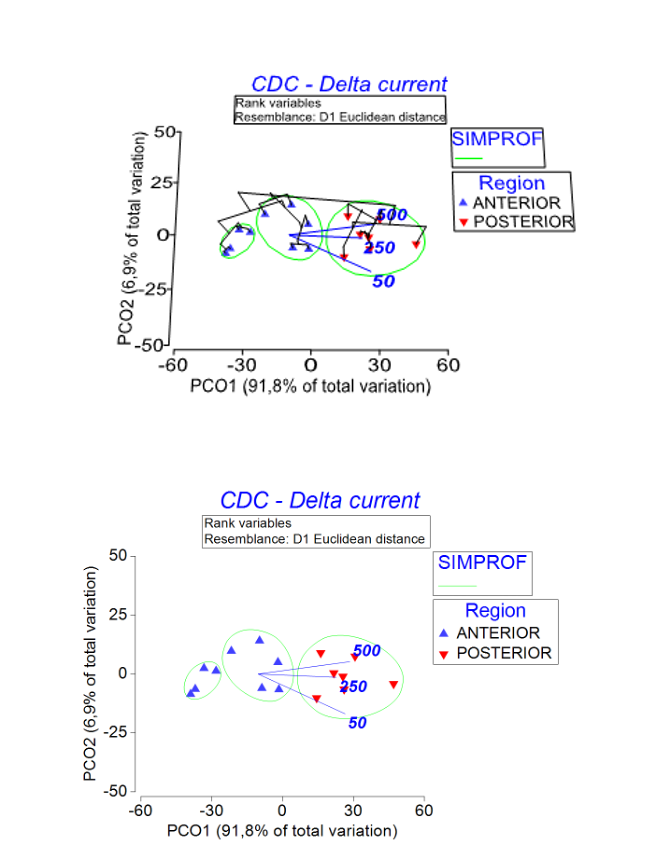


**Supplementary figure 1**. Principal Coordinate (PCO) analysis plot, showing the first two principal coordinates that, combined, explain 98.7% of the total variance observed in the distance matrix (D1 Euclidian distances) between delta current values measured in anterior and posterior intestine samples, incubated with CDC (50, 250 and 500 μg/ml). An analysis of similarities (ANOSIM), testing for differences between unordered intestinal region groups, showed a sample statistic (R) of 0.849 with a significance level of sample statistics of 0.001.

The groups of samples indicated were obtained with a SIMPROF analysis and showed 95% confidence intervals, further supporting the cluster analysis, which had a cophenetic correlation of 0.76.


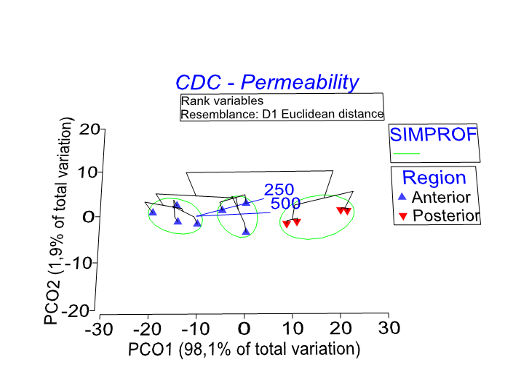


**Supplementary figure 2**. Principal Coordinate (PCO) analysis plot, showing the first two principal coordinates that, combined, explain 100% of the total variance observed in the distance matrix (D1 Euclidian distances) between permeability values measured in anterior and posterior intestine samples, incubated with CDC (250 and 500 μg/ml). An analysis of similarities (ANOSIM), testing for differences between unordered intestinal region groups, showed a sample statistic (R) of 0.835 with a significance level of sample statistics of 0.003.

The groups of samples indicated were obtained with a SIMPROF analysis and showed 95% confidence intervals, further supporting the cluster analysis, which had a cophenetic correlation of 0.76.


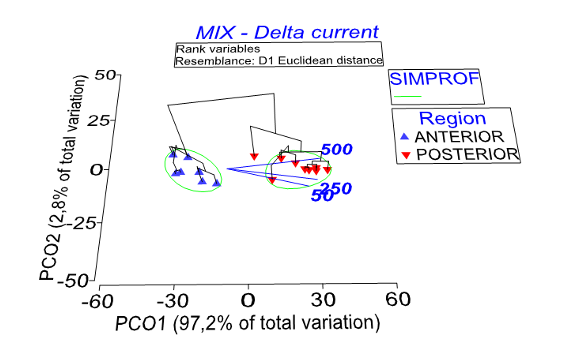


**Supplementary figure 3**. Principal Coordinate (PCO) analysis plot, showing the first two principal coordinates that, combined, explain 100% of the total variance observed in the distance matrix (D1 Euclidian distances) between delta current values, measured in anterior and posterior intestine samples, incubated with MIX (50, 250 and 500 μg/ml). An analysis of similarities (ANOSIM), testing for differences between unordered intestinal region groups, showed a sample statistic (R) of 0.987 with a significance level of sample statistics of 0.001.

The groups of samples indicated were obtained with a SIMPROF analysis and showed 95% confidence intervals, further supporting the cluster analysis, which had a cophenetic correlation of 0.90.
